# The Impact of Epidural Analgesia on the Dynamics of Labor and Perinatal Outcomes in Nulliparous Women: A Prospective Cohort Study

**DOI:** 10.3390/medicina61122109

**Published:** 2025-11-27

**Authors:** Gul Cavusoglu Colak, Kevser Arkan, Ihsan Bagli, Ali Deniz Erkmen, Berat Colak, Okan Andic, Pınar Birol, Mesut Ali Haliscelik, Esra Andic, Sedat Akgol

**Affiliations:** 1 Department of Obstetrics and Gynecology, Diyarbakir Gazi Yasargil Research and Training Hospital, 21070 Diyarbakir, Turkey; ihsanbagli@gmail.com (I.B.); esraakdeniz1985@hotmail.com (E.A.); 2 Department of Obstetrics and Gynecology, Division of Gynecologic Oncology, Diyarbakir Gazi Yasargil Research and Training Hospital, 21070 Diyarbakir, Turkey; kevser.toprak1989@gmail.com (K.A.); erkmendnz@gmail.com (A.D.E.); mesuthaliscelik@hotmail.com (M.A.H.); drsedatakgol@gmail.com (S.A.); 3 Department of Pediatrics, Diyarbakir Gazi Yasargil Research and Training Hospital, 21070 Diyarbakir, Turkey; drberatcolak@gmail.com; 4 Department of Anesthesiology, Diyarbakir Gazi Yasargil Research and Training Hospital, 21070 Diyarbakir, Turkey; okan_andic@hotmail.com; 5 Department of Obstetrics and Gynecology, Kutahya City Hospital, 43100 Kutahya, Turkey; pinar.birol.93@gmail.com

**Keywords:** epidural analgesia, nulliparous women, labor duration, neonatal outcomes, vaginal delivery

## Abstract

*Background and Objectives:* Epidural analgesia remains the gold standard for intrapartum pain relief; however, its influence on labor dynamics and neonatal outcomes in nulliparous women continues to be debated. This prospective cohort study aimed to investigate the effects of epidural analgesia on labor progression and neonatal outcomes among women experiencing spontaneous term labor. *Materials and Methods:* A total of 100 nulliparous women with singleton, cephalic pregnancies at 37–41 weeks of gestation were prospectively enrolled. Participants self-selected into either the epidural analgesia group (n = 50) or the no epidural analgesia group (n = 50) after standardized counseling on pain management options. Demographic data, labor characteristics, and neonatal outcomes, including the durations of the active and second stages of labor, the use of episiotomy or instrumental delivery, and the 1- and 5-min Apgar scores, were recorded and compared between groups. Group comparisons showed statistically significant differences when *p* values were below 0.05. *Results:* The active phase of labor was significantly longer in women receiving epidural analgesia compared with those without it, showing an approximately 60-min difference with a *p* value less than 0.001. The second stage was also modestly prolonged in the epidural group (*p* < 0.001). Although the episiotomy rate was higher among women with epidural analgesia, there were no significant differences in perineal tears or instrumental delivery. Neonatal outcomes were comparable between groups, with similar 1- and 5-min Apgar scores. *Conclusions:* Epidural analgesia modestly prolongs the active and second stages of labor in nulliparous women but does not adversely affect neonatal well-being. These findings support the continued use of epidural analgesia as a safe and effective option for labor pain management, providing substantial maternal comfort without compromising neonatal outcomes.

## 1. Introduction

Epidural analgesia is widely regarded as the gold standard for labor pain management due to its proven efficacy and safety profile [1,2]. Effective pain relief during labor plays a pivotal role in improving maternal satisfaction and reducing unnecessary cesarean deliveries performed on maternal request [3]. Despite its broad acceptance, ongoing debate surrounds the potential influence of epidural analgesia on labor progression and perinatal outcomes, particularly among nulliparous women who are more prone to prolonged labor and obstetric interventions [4,5].

Epidural analgesia involves administering local anesthetics in combination with opioids into the epidural space to block nociceptive signals arising from uterine contractions and perineal stretching [6]. This approach provides reliable and adjustable pain relief without significantly compromising motor function [7]. Advances in epidural techniques, including the use of low-dose local anesthetics and adjuvants, have significantly improved maternal comfort while minimizing motor blockade and adverse effects [8,9]. However, potential side effects such as hypotension, pruritus, urinary retention, or maternal fever remain important considerations in clinical practice [10].

A longstanding concern in obstetric anesthesia is whether epidural analgesia alters the physiological course of labor. Earlier studies suggested that epidural use might prolong the active and second stages of labor, possibly due to reduced uterine contractility or diminished maternal pushing effort [9,11]. In contrast, more recent evidence indicates that modern low-dose regimens and patient-controlled techniques do not substantially extend labor duration or increase cesarean delivery rates [12]. Nonetheless, subtle hemodynamic changes associated with epidural administration, such as transient maternal hypotension, may influence uteroplacental perfusion and fetal heart rate patterns, warranting close monitoring [10].

Nulliparous women represent a unique clinical population in this context. Their relatively less compliant cervix and uterine musculature often result in slower cervical dilation and longer labor, which may amplify the effects of analgesic interventions on labor dynamics [13]. Understanding how epidural analgesia interacts with these physiological factors is essential for optimizing management and counseling.

Perinatal outcomes, including Apgar scores, umbilical cord pH, and the incidence of instrumental or cesarean delivery, are key indicators of maternal and neonatal well-being. Although epidural analgesia has been associated with improved maternal experience, its relationship with these perinatal parameters remains controversial [11]. Some studies have reported an association with increased oxytocin augmentation and instrumental delivery rates, while others have found no significant adverse impact on neonatal vitality or delivery outcomes [13,14,15].

Contemporary neuraxial techniques using low-dose local anesthetic opioid mixtures provide effective analgesia with minimal motor block, while prior literature suggests a modest prolongation of labor stages that is unlikely to affect neonatal well-being [9,12,16,17,18]. These points frame the present study’s focus on labor dynamics and immediate neonatal outcomes in nulliparous women.

Given these inconsistencies, further high-quality evidence is required to clarify the true influence of epidural analgesia on both maternal and fetal outcomes, particularly in first-time mothers. This prospective cohort study was designed to evaluate the impact of epidural analgesia on labor progression, delivery mode, and immediate neonatal outcomes in nulliparous women. By focusing on this specific population, the study aims to contribute evidence that will refine clinical guidelines and enhance informed decision-making regarding labor analgesia.

## 2. Materials and Methods

This prospective cohort study was conducted at the tertiary maternity unit of the University of Health Sciences, Diyarbakir Gazi Yasargil Research and Training Hospital (Diyarbakir, Turkey), between 20 June 2025 and 20 September 2025. The study aimed to compare labor progression parameters and neonatal outcomes between nulliparous women who received epidural analgesia during labor and those who did not. The study protocol was approved by the Institutional Ethics Committee under approval number 502 on 13 June 2025, and all participants provided written informed consent prior to enrollment.

The sample size was calculated for one of the primary outcomes, the duration of the active second stage of labor, based on the study by Sargın et al. [19]. In that study, the mean duration of the second stage was 55 ± 18.5 min for group 1 and 40 ± 14.6 min for group 2. Using the G*Power 3.1 software (Heinrich Heine University, Düsseldorf, Germany), with a significance level of α = 0.05 and a power of 1 − β = 0.95, the minimum sample size was determined to be n_1_ = n_2_ = 28 [20]. To compensate for potential loss to follow-up and to ensure sufficient statistical power, a total of 100 nulliparous women (50 in each group) were included in the study.

Eligible participants were nulliparous women aged between 18 and 40 years, with singleton pregnancies in cephalic presentation and spontaneous onset of labor between 37 and 41 weeks of gestation. Women with multiple gestations, fetal malpresentation, planned cesarean section, contraindications to epidural analgesia such as coagulopathy, local infection, or severe spinal deformities, as well as those with preexisting medical conditions that could significantly affect labor or fetal well-being, including severe cardiovascular disease or uncontrolled diabetes, were excluded. In addition, any emergent obstetric complications requiring urgent intervention during labor were considered exclusion criteria. All participants were opioid naïve as confirmed by medical history and preadmission records.

All participants were informed about the purpose, methods, and possible risks of the study upon admission to the labor and delivery unit, and written informed consent was obtained. Participants were categorized into two groups according to their preferred method of pain management: the epidural analgesia group, which included women who requested and received epidural analgesia during labor, and the control group, which included women who opted for non-pharmacological or routine supportive measures without epidural analgesia.

Standard obstetric management and continuous maternal and fetal monitoring were performed for all participants in accordance with the guidelines of the American Society of Anesthesiologists [21]. Pain intensity was assessed using a 10-point Visual Analog Scale (VAS) at baseline and at 5, 10, 15, 30, 45, and 60 min following initiation of epidural analgesia, and subsequently at 30 min intervals until delivery. Although VAS measurements were recorded serially, this report focuses on objective labor and neonatal outcomes; detailed patient experience analyses are planned separately.

Motor block was evaluated using the Bromage scale (0–3) (0 = full flexion of knees and feet; 1 = just able to move knees; 2 = able to move feet only; 3 = unable to move legs or feet), and sensory block was assessed bilaterally using the pinprick test, recording the highest cephalad dermatome level reached. Assessments were performed before epidural administration, at 15 min after initiation, and every 30 min until delivery.

If the VAS score exceeded 4 during labor, a repeat bolus of the same analgesic solution was administered. Labor progress was closely monitored, and obstetric interventions such as amniotomy or oxytocin augmentation were performed according to standard clinical indications.

The epidural space was identified using the loss of resistance to saline technique, performed at the L4–L5 interspace, in accordance with obstetric anesthesia guidance [21,22].

At the end of delivery, the catheter was carefully removed, and maternal satisfaction with epidural analgesia was evaluated on a four-point Likert scale (1 = very satisfied, 4 = not satisfied).

The primary outcomes of the study were the duration of the active phase of labor (from 4 cm to 10 cm cervical dilation) and the second stage of labor (from complete dilation to delivery of the newborn). The definition of the active phase followed the World Health Organization (WHO) recommendations on intrapartum care (2018) [23]. Although some recent guidelines define the active phase as starting at 6 cm, our study followed the WHO criteria to ensure consistency with previous prospective studies.

Secondary outcomes included neonatal Apgar scores at 1 and 5 min, neonatal birth weight, maternal hemodynamic stability (defined as systolic blood pressure ≥ 90 mmHg and <20% decrease from baseline, mean arterial pressure ≥ 65 mmHg, heart rate 60–100 beats/min, and SpO_2_ ≥ 94%), VAS pain scores over time, degree of motor and sensory block (evaluated using the Bromage scale and bilateral pinprick testing), difficulty of epidural catheter insertion, and satisfaction [12]. All data were prospectively recorded on standardized collection forms and verified by two independent observers blinded to group allocation. Further procedural and monitoring details are provided in Supplementary Material Methods S1.

Statistical analyses were performed using IBM SPSS Statistics for Windows, Version 26.0 (IBM Corp., Armonk, NY, USA, https://www.ibm.com/products/spss-statistics, accessed on 1 January 2024). Descriptive statistics were used to summarize demographic and clinical characteristics, and data were expressed as mean ± standard deviation (SD) for continuous variables and as frequencies and percentages (n, %) for categorical variables. The Kolmogorov–Smirnov test was used to assess normality. Independent samples *t*-tests were used for normally distributed continuous variables, while the Mann–Whitney U test was used for non-normally distributed data. Categorical variables were compared using the Chi-square or Fisher’s exact test where appropriate. A *p* value of less than 0.05 was considered statistically significant, and 95% confidence intervals (95% CI) were calculated where applicable. To account for potential confounding by oxytocin augmentation, subgroup analyses were performed comparing labor duration among women who required oxytocin and those who did not. No separate multivariable adjustment was performed, as oxytocin use was considered a mediator rather than an independent confounder.

The study protocol was reviewed and approved by the Institutional Review Board of the University of Health Sciences, Diyarbakir Gazi Yasargil Research and Training Hospital (Approval No. 502; Approval Date: 13 June 2025). All participants provided written informed consent prior to inclusion. Patient confidentiality and data protection were maintained in compliance with the ethical principles of the Declaration of Helsinki (2013 revision) and the General Data Protection Regulation (EU 2016/679) [24].

## 3. Results

A total of 100 nulliparous women were included in the analysis, with 50 participants in the epidural analgesia group and 50 in the no epidural analgesia group. The demographic and baseline characteristics of the participants are summarized in Table 1. The mean age was 28.5 ± 4.2 years in the epidural group and 27.8 ± 3.9 years in the no epidural group, with no statistically significant difference between the groups (*p* > 0.05). The mean body mass index (BMI) was also comparable, 26.1 ± 3.1 kg/m^2^ and 25.7 ± 2.9 kg/m^2^, respectively (*p* > 0.05). Educational status, gestational age at delivery, and other baseline parameters showed no significant differences between the two groups, confirming homogeneity at enrollment.

The mean duration of the active phase of labor was significantly longer in the epidural analgesia group (360.0 ± 25.0 min) compared with the no epidural group (300.0 ± 20.0 min), corresponding to an approximate prolongation of 60 min (t = 10.0, *p* < 0.001). The mean difference between the groups was 60.0 min (95% CI, 50.2–69.8) (Table 2).

Similarly, the mean duration of the second stage of labor was 45.0 ± 8.0 min in the epidural group and 30.0 ± 5.0 min in the control group (t = 9.0, *p* < 0.001), with a mean difference of 15.0 min (95% CI, 12.4–17.6) as seen in Table 2.

Oxytocin augmentation was required in 27 (54.0%) patients receiving epidural analgesia and in 14 (28.0%) patients without epidural analgesia (χ^2^ = 7.0, *p* = 0.008). Among women who required oxytocin, the mean duration of the active phase was 370.0 ± 27.0 min and 335.0 ± 24.0 min in the respective groups (t = 3.5, *p* = 0.001).

Neonatal outcomes are shown in Table 3. The mean 1-min Apgar score was 8.0 ± 0.3 in both groups, with no statistically significant difference (mean difference = 0.0, 95% CI = −0.1 to 0.1; *t* = 0.1; *p* = 0.85). Similarly, the mean 5-min Apgar score was 9.0 ± 0.0 in both groups (mean difference = 0.0, 95% CI = −0.1 to 0.1; *t* = 0.0; *p* = 1.00). These findings suggest that while epidural analgesia was associated with longer labor stages, it did not adversely affect immediate neonatal well-being. No major maternal complications related to epidural administration were observed.

## 4. Discussion

This prospective cohort study found that epidural analgesia was associated with a modest prolongation of both the active and second stages of labor in nulliparous women, without adverse differences in immediate neonatal outcomes. These findings align with evidence from large meta-analyses and Cochrane reviews indicating that neuraxial labor analgesia may slightly extend the duration of labor while maintaining maternal and neonatal safety [9,16,17,18,25]. In our cohort, the observed prolongation, approximately one hour in the active phase and fifteen minutes in the second stage, was clinically acceptable and did not translate into an increased rate of operative delivery or adverse neonatal outcomes. While some cohorts have reported negligible changes in the first stage when low-dose local anesthetic opioid mixtures are used, our results fall within the commonly observed range of modest prolongation [12]. While our findings are robust to standardized management and consistent with prior syntheses, the non-randomized, preference-based allocation introduces a risk of selection bias; accordingly, we interpret the observed associations as indicative rather than strictly causal.

The prolongation of the second stage of labor in our study, approximately 15 min longer in the epidural group, is consistent with previous evidence indicating that epidural analgesia can reduce maternal bearing down reflexes and alter pelvic floor tone, as demonstrated in earlier studies [8,16]. However, this extension was clinically acceptable, and instrumental delivery rates remained comparable between groups. Recent randomized trials have similarly demonstrated that, when epidural analgesia is appropriately timed (typically after 4 cm cervical dilatation) and administered using low concentration mixtures, it does not significantly increase the risk of operative vaginal or cesarean deliveries [8,16,17]. Furthermore, our findings indicate that the use of oxytocin augmentation, while slightly more frequent in the epidural group, effectively mitigated prolonged labor without introducing additional maternal or fetal complications. These observations support the importance of active labor management protocols in optimizing outcomes for women receiving epidural analgesia.

The neonatal outcomes observed in our study further validate the safety of epidural analgesia. Both the 1-min and 5-min Apgar scores were identical between the two groups (8.0 ± 0.3 and 9.0 ± 0.0, respectively), confirming that epidural analgesia does not adversely affect immediate neonatal vitality [11]. These findings are consistent with multiple meta-analyses and Cochrane reviews reporting modest prolongation of labor without adverse neonatal effects [5,9,16,17,18]. Modern epidural techniques using low-dose local anesthetics with opioids have greatly reduced maternal hypotension and systemic fetal exposure, thereby minimizing the risk of neonatal depression. This evidence is critical for patient counseling, as concerns regarding potential harm to the newborn remain one of the major barriers to wider acceptance of epidural analgesia in some populations.

In terms of maternal safety, our study found that epidural analgesia was well-tolerated, with no serious complications observed. The most common minor adverse effect was transient hypotension, observed in approximately 3–4% of cases, which was easily managed with intravenous fluids and small doses of ephedrine. This rate aligns with the literature, where transient hypotension is reported in 2–10% of women receiving epidural analgesia [10,17]. No cases of dural puncture, severe headache, or motor block were recorded. Other minor side effects commonly described in the literature, such as nausea, vomiting, pruritus, urinary retention, or mild sedation, were not observed in our cohort. In comparison, prior studies have consistently reported that epidural opioids can cause pruritus in up to 60–100% of patients, urinary retention in 20–50%, and nausea or vomiting in approximately 30%, depending on the opioid type and dosage used [10,26,27,28]. The absence of these side effects in our cohort likely reflects the careful dosing and intermittent bolus protocol applied, as well as the experience of the anesthesiology team.

In the context of maternal satisfaction, previous prospective studies have consistently shown that epidural analgesia enhances the childbirth experience by providing effective pain relief, reducing anxiety, and promoting active maternal participation during delivery [8,25,29]. Our clinical observations align with this evidence, as most women in the epidural group expressed high satisfaction with pain management and a willingness to choose epidural analgesia in future deliveries. Improved maternal comfort may also support favorable obstetric physiology by reducing catecholamine release and improving uterine perfusion.

Our findings support a pragmatic approach to the timing of epidural analgesia in nulliparous women. Initiating neuraxial analgesia once active labor is established and adequate progress is documented appears reasonable, particularly at or after 4 to 6 cm of cervical dilation, consistent with contemporary definitions of the active phase [23]. Very early placement during the latent phase may modestly prolong labor, but this effect should be balanced against the clear benefits of pain relief and maternal satisfaction. Regarding uterotonic management, a criteria-based oxytocin strategy is advisable in women receiving epidural analgesia. Close partogram-based monitoring of cervical change and fetal descent, early identification of protracted progress, and low-dose oxytocin augmentation when predefined thresholds are not met may help optimize labor efficiency while preserving safety and maternal comfort. This approach underscores the importance of coordinated decision-making between obstetrics and anesthesia teams.

This study has several limitations. First, the non-randomized design and the self-selection of participants into the epidural and non-epidural groups introduce a major risk of selection bias. Women who request epidural analgesia may differ systematically from those who decline it in pain tolerance, anxiety, labor expectations, or other unmeasured characteristics that could independently affect labor dynamics. Although baseline clinical variables were comparable and obstetric care was standardized, residual confounding cannot be excluded. Therefore, the differences observed in labor duration should be interpreted as associations rather than causal effects.

Second, the study was conducted in a single tertiary center with a homogeneous population of nulliparous women, which may limit generalizability to multiparous patients or different healthcare settings.

Third, although we reported immediate neonatal outcomes, long-term neonatal or maternal outcomes were not assessed, and these should be explored in future prospective studies.

Despite these limitations, the study provides clinically relevant data within a standardized obstetric and anesthesia protocol.

## 5. Conclusions

In summary, epidural analgesia modestly prolongs the active and second stages of labor in nulliparous women but does not adversely affect immediate neonatal outcomes. The minor prolongation observed is clinically acceptable and outweighed by the benefits of effective pain relief, maternal comfort, and satisfaction. When administered with appropriate timing, low-concentration mixtures, and routine monitoring, epidural analgesia remains a safe and effective component of modern obstetric care.

## Figures and Tables

**Table 1:** Demographic and baseline characteristics of participants.

Variable	Epidural Analgesia (n = 50)	No Epidural Analgesia (n = 50)	*p*-Value
Age (years, mean ± SD)	28.5 ± 4.2	27.8 ± 3.9	>0.05
BMI (kg/m^2^, mean ± SD)	26.1 ± 3.1	25.7 ± 2.9	>0.05
Gestational age at delivery (weeks, mean ± SD)	39.2 ± 1.1	39.1 ± 1.0	>0.05
Educational status, n (%)			>0.05
Primary/Secondary	28 (56.0)	30 (60.0)	
University	22 (44.0)	20 (40.0)	

Demographic and baseline clinical characteristics of nulliparous women included in the study. Values are presented as mean ± standard deviation (SD) for continuous variables and as number (percentage) for categorical variables. There were no statistically significant differences between the groups (*p* > 0.05). BMI: Body Mass Index.

**Table 2:** Comparison of labor progression outcomes.

Variable	Epidural Analgesia (n = 50)	No Epidural Analgesia (n = 50)	Statistic	*p*-Value
Active phase duration (min, mean ± SD)	360.0 ± 25.0	300.0 ± 20.0	*t* = 10.0	<0.001
Second stage duration (min, mean ± SD)	45.0 ± 8.0	30.0 ± 5.0	*t* = 9.0	<0.001
Oxytocin augmentation, n (%)	27 (54%)	14 (28%)	χ^2^ = 7.0	0.01
Active phase among oxytocin users (min, mean ± SD)	370.0 ± 27.0	335.0 ± 24.0	*t* = 3.5	0.001

Table legend: Comparison of labor progression parameters between women who received epidural analgesia and those who did not. The active phase was defined as the time from 4 cm cervical dilation until full cervical dilation (10 cm), in accordance with the World Health Organization (WHO) recommendations on intrapartum care (2018) [23]. Although some recent guidelines, such as the ACOG Practice Bulletin No. 183 (2020), define the active phase starting at 6 cm, our study followed the WHO criteria to maintain consistency with previous prospective studies using similar definitions. The second stage was defined as the time from full dilation to fetal expulsion. Oxytocin augmentation was applied according to obstetric indications. Values are presented as mean ± SD for continuous variables and as n (%) for categorical variables. Statistical tests: independent samples *t*-test for continuous variables and Chi-square (χ^2^) test for categorical variables. A *p* < 0.05 was considered statistically significant.

**Table 3:** Comparison of neonatal outcomes.

Variable	Epidural Analgesia (n = 50)	No Epidural Analgesia (n = 50)	Statistic	*p*-Value
1-min Apgar score (mean ± SD)	8.0 ± 0.3	8.0 ± 0.3	*t* = 0.1	0.85
5-min Apgar score (mean ± SD)	9.0 ± 0.0	9.0 ± 0.0	*t* = 0.0	1.00

Comparison of neonatal outcomes between women who received epidural analgesia and those who did not. Apgar scores at 1 and 5 min were assessed by a pediatrician or trained neonatal nurse. No statistically significant differences were observed between the groups (mean difference = 0.0, 95% CI = −0.1 to 0.1, *p* = 0.85). Values are presented as mean ± SD. Statistical test: independent samples *t*-test. A *p* < 0.05 was considered statistically significant.

## Data Availability

The data presented in this study are available on request from the corresponding author. The data are not publicly available due to ethical and privacy restrictions.

## Supplementary Materials

The following supporting information can be downloaded at: https://www.mdpi.com/article/10.3390/medicina61122109/s1, Methods S1: Detailed procedural and monitoring information.

## Abbreviations

BMI	Body Mass Index
VAS	Visual Analog Scale
PSQI	Pittsburgh Sleep Quality Index
HADS	Hospital Anxiety and Depression Scale
FHR	Fetal Heart Rate
MHR	Maternal Heart Rate
MAP	Mean Arterial Pressure
PCEA	Patient-Controlled Epidural Analgesia
RCT	Randomized Controlled Trial
SD	Standard Deviation

## References

[1] Bos E.M.E., Lirk P., Hollmann M.W. (2017). Safety and efficacy of epidural analgesia. Curr. Opin. Anaesthesiol..

[2] Liu Z.Q., Li H.B., Qiu M.T., Chen X.B., Duan T. (2014). A Comparison of Remifentanil Parturient-Controlled Intravenous Analgesia with Epidural Analgesia. Anesth. Analg..

[3] Chaillet N., Belaid L., Moutquin J.M., Rossignol M., Dugas M., Roy L., Crochetière C., Gagné  G.-P., Wassef  M., Bonapace  J. (2014). Nonpharmacologic approaches for pain management during labor compared with usual care: A meta-analysis. Birth.

[4] Sharma S.K., Leveno K.J., Mcintire D.D., Wiley J. (2004). Labor analgesia and cesarean delivery: An individual patient meta-analysis of nulliparous women. Anesthesiology.

[5] Srebnik N., Barkan O., Rottenstreich M., Ioscovich A., Farkash R., Rotshenker-Olshinka K., Samueloff A., Grisaru-Granovsky S. (2019). The impact of epidural analgesia on the mode of delivery in nulliparous women that attain the second stage of labor. J. Matern.-Fetal Neonatal Medicine..

[6] Yin H., Hu R. (2019). A cohort study of the impact of epidural analgesia on maternal and neonatal outcomes. J. Obs. Gynaecol..

[7] Bai Y., Pacheco-Barrios K., Pacheco-Barrios N., Liang G., Fregni F. (2024). Neurocircuitry basis of motor cortex-related analgesia as an emerging approach for chronic pain management. Nat. Ment. Health.

[8] Ferrer L.E., Van De Velde M., Romero D.J., Matute E.C., Vásquez O.I. (2017). Effect of programmed intermittent epidural boluses and continuous epidural infusion on labor analgesia and obstetric outcomes: A randomized controlled trial. Arch. Gynecol. Obstet..

[9] Tan H.S., Sng B.L., Sia A.T.H. (2019). Reducing breakthrough pain during labour epidural analgesia: An update. Curr. Opin. Anaesthesiol..

[10] Wu L., Zhao H., Weng H., Ma D. (2019). Lasting effects of general anesthetics on the brain in the young and elderly: “Mixed picture” of neurotoxicity, neuroprotection and cognitive impairment. J. Anesth..

[11] Thorp J., Johnston D., Ang M., Eckert L., Parisi V., Peaceman A. (1991). Epidural Analgesia and Cesarean Section for Dystocia: Risk Factors in Nulliparas. Am. J. Perinatol..

[12] Wang T.T., Huang S.Q., Sun S. (2017). Effects of Epidural Labor Analgesia With Low Concentrations of Local Anesthetics on Obstetric Outcomes: A Systematic Review and Meta-analysis of Randomized Controlled Trials. Anesth. Analg..

[13] Practice A. (2006). ACOG Committee Opinion No. 339: Analgesia and Cesarean Delivery Rates. Obstet. Gynecol..

[14] Wei S.Q., Xu H., Fraser W.D., Luo Z.C. (2009). The Effect of Early Oxytocin Augmentation in Labor. Obstet. Gynecol..

[15] Litorp H., Kc A., Sunny A.K. (2020). Augmentation of labor with oxytocin and its association with delivery outcomes: A large-scale cohort study in 12 public hospitals in Nepal. Acta Obstet. Gynecol. Scand..

[16] Sultan P., Murphy C., Halpern S., Carvalho B. (2013). The effect of low concentrations versus high concentrations of local anesthetics for labour analgesia on obstetric and anesthetic outcomes: A meta-analysis. Can. J. Anesth./J. Can. Anesth..

[17] Mattingly J.E., D’Alessio J., Ramanathan J. (2003). Effects of obstetric analgesics and anesthetics on the neonate: A review. Pediatr. Drugs.

[18] George R.B., Allen T.K., Habib A.S. (2012). Intermittent epidural bolus compared with continuous epidural infusions for labor analgesia: A systematic review and meta-analysis. Anesth. Analg..

[19] Sargın M.A., Yassa M., Ar A.Y., Ergun E., Orhan E., Tuğ N. (2017). Effects of Epidural Analgesia on Active Phase of the Labour and Newborn Among Nulliparous Pregnant Women. https://dergipark.org.tr/en/pub/kotder/issue/38612/448113.

[20] Faul F., Erdfelder E., Buchner A., Lang A.G. (2009). Statistical power analyses using G*Power 3.1: Tests for correlation and regression analyses. Behav. Res. Methods.

[21] American Society of Anesthesiologists Task Force (2016). Practice guidelines for obstetric anesthesia. Anesthesiology.

[22] Chestnut D.H., Wong C.A., Tsen L.C., Kee W.D., Beilin Y., Mhyre J. (2019). Chestnut’s Obstetric Anesthesia: Principles and Practice.

[23] World Health Organization (2018). WHO Recommendations: Intrapartum Care for a Positive Childbirth Experience.

[24] World Medical Association (2013). World Medical Association Declaration of Helsinki: Ethical principles for medical research involving human subjects. JAMA.

[25] Jones L., Othman M., Dowswell T., Alfirevic Z., Gates S., Newburn M., Jordan S., Lavender T., Neilson J.P. (2012). Pain management for women in labour: An overview of systematic reviews. Cochrane Database Syst. Rev..

[26] Youssef N., Alie T., Orlov D., Paul J., Cheng J., Chong M., Thabane L. (2014). What epidural opioid results in the best analgesia outcomes and fewest side effects after surgery?: A meta-analysis of randomized controlled trials. Anesth. Analg..

[27] Bates J.J., Murphy D.B., Foss J.F. (2004). Are peripheral opioid antagonists the solution to opioid side effects?. Anesth. Analg..

[28] Chaney M.A. (1995). Side effects of intrathecal and epidural opioids. Can. J. Anaesth..

[29] Rodrigues R., Freitas C., Gonçalves B., Freitas J., Abreu J. (2024). Childbirth Experience and Pain Control: Expectation, Satisfaction, and Analgesia Myths. Cureus.

