# Supplementary material for: The Impact of Epidural Analgesia on the Dynamics of Labor and Perinatal Outcomes in Nulliparous Women: A Prospective Cohort Study
Source: Medicina (Kaunas). 2025 Nov 27;61(12):2109. doi: 10.3390/medicina61122109 (PMC12734504; doi:10.3390/medicina61122109)
Supplement: Supplementary file 1 [file medicina-61-02109-s001.zip › medicina-3954902-supplementary.pdf]

Supplementary Methods S1. Detailed procedural and monitoring information

1. Hemodynamic Monitoring Protocol

Maternal hemodynamic stability was predefined as systolic blood pressure (SBP) ≥ 90 mmHg and < 20% decrease from baseline, mean arterial pressure (MAP) ≥ 65 mmHg, heart rate (HR) between 60-100 beats per minute, and SpO_2 ≥ 94% on room air. Measurements were recorded every 5 minutes for the first 30 minutes after epidural initiation and every 15-30 minutes thereafter until delivery. Any deviation from these thresholds lasting ≥ 1 minute was defined as a hemodynamic instability episode. Hypotension was treated with 5 mg intravenous ephedrine as required, and both the number of hypotension episodes and total vasopressor dose were recorded.

2. Epidural Analgesia Procedure and Dosing Parameters

For the epidural analgesia group, patients were placed in the left lateral or sitting position, and after aseptic skin preparation, local anesthesia was achieved using 2 mL of 2% lidocaine (40 mg) at the L4-L5 intervertebral space.

An initial bolus dose of 10-15 mL of 0.125% bupivacaine with 2 μg/mL fentanyl was administered, followed by continuous infusion or patient controlled epidural analgesia (PCEA) using 0.0625% bupivacaine with 2 μg/mL fentanyl, in accordance with the institutional obstetric anesthesia protocol of the University of Health Sciences, Diyarbakir Gazi Yasargil Research and Training Hospital, which adheres to the national guidelines of the Turkish Society of Anesthesiology and Reanimation (2023) and the ASA Practice Guidelines for Obstetric Anesthesia [24,38].

Among women receiving epidural analgesia, 22 (44%) received continuous infusion, 18 (36%) received intermittent bolus, and 10 (20%) received PCEA. For PCEA, a standardized protocol was used: an on-demand bolus of 5 mL of 0.0625% bupivacaine with 2 μg/mL fentanyl, with a 15-minute lockout interval and a maximum hourly limit of 20 mL. These parameters were identical for all patients and followed the institutional obstetric anesthesia guideline.

3. Sensory and Motor Block Assessment

Motor block was evaluated using the Bromage scale (0–3) (0 = full flexion of knees and feet; 1 = just able to move knees; 2 = able to move feet only; 3 = unable to move legs or feet), and sensory block was assessed bilaterally using the pinprick test, recording the highest cephalad dermatome level reached. Assessments were performed before epidural administration, at 15 minutes after initiation, and every 30 minutes until delivery.

4. Catheter Placement Difficulty Scale

The difficulty of epidural catheter insertion was graded on a four-point scale (0 = easy, 1 = moderate, 2 = difficult, 3 = very difficult), and all epidural procedures were performed by the same anesthesiology specialist to minimize variability.

Maternal hemodynamics and fetal heart rate were continuously monitored before, during, and

after the procedure. If maternal hypotension or side effects such as nausea, vomiting, pruritus, or motor weakness occurred, appropriate pharmacologic interventions were administered and documented.
